# Prognostic Significance of DSCC1, a Biomarker Associated with Aggressive Features of Breast Cancer

**DOI:** 10.3390/medicina60121929

**Published:** 2024-11-23

**Authors:** Abrar I. Aljohani

**Affiliations:** Department of Clinical Laboratory Sciences, College of Applied Medical Sciences, Taif University, Taif 21944, Saudi Arabia; abrar.g@tu.edu.sa; Tel.: +966-9200-02122

**Keywords:** DSSC1, invasive breast cancer, prognosis

## Abstract

*Background and Objectives*: Invasive breast cancer (BC) was traditionally investigated visually, and no technique could identify the key molecular drivers of patient survival. However, essential molecular drivers of invasive BC have now been discovered using innovative genomic, transcriptomic, and proteomic methodologies. Nevertheless, few evaluations of the prognostic factors of BC in Saudi Arabia have been performed. Evaluating the biomarkers associated with the development of early-stage BC could help determine the risk of metastasis and guide treatment decisions. In a previous study, using large BC cohorts and artificial neural network techniques, DNA replication and sister chromatid cohesion 1 (*DSCC1*) was found to be one of the principal genes in invasive BC samples. To date, no studies have addressed the prognostic significance of *DSCC1* in invasive BC and its association with aggressive tumor behavior. This research aimed to address this gap. *Materials and Methods*: The association of clinicopathological features and patient outcomes with *DSCC1* expression at the mRNA level was assessed using the Molecular Taxonomy Breast Cancer International Consortium (METABRIC; *n* = 1980) and The Cancer Genome Atlas (TCGA; *n* = 854) cohorts. *DSCC1* was also evaluated at the protein level using immunohistochemistry on samples from invasive BC patients (*n* = 100) presenting to King Abdul Aziz Specialist Hospital in Saudi Arabia. The association of clinicopathological parameters (including patient age, tumor grade, tumor size, and patient outcome) with protein level was also evaluated. *Results*: In both METABRIC and TCGA cohorts, high expression of *DSCC1* was significantly associated with high histological grade, large tumor size, lymphovascular invasion positivity, and hormone receptor negativity (all *p* < 0.001). A high *DSCC1* mRNA level was associated with poor outcomes (*p* < 0.001 for METABRIC, *p* = 0.23 for TCGA). At the protein level, high DSCC1 expression was associated with high histological grade (*p* = 0.001), lymph node presence (*p* = 0.008), hormone receptor negativity (*p* = 0.005), high Ki67 expression (*p* = 0.036), and shorter survival (*p* = 0.008). *Conclusions*: This study confirmed the prognostic significance of DSCC1 in invasive BC patients. DSCC1 could be a therapeutic target in BC cases with poor outcomes.

## 1. Introduction

Breast cancer (BC) remains the most diagnosed and complex disease and the main cause of cancer-related mortality in women globally [1]. Inter-tumor and intra-tumor heterogeneity suggest that individual cases of BC may respond differently to treatment and have different prognoses. Since BC is a dynamic disease that changes as the tumor grows and spreads, the viability of a customized therapeutic strategy is fundamentally challenged [2]. In clinical practice, prognostic or predictive factors can be utilized to predict outcomes and guide the selection of systemic therapy [3]. Several prognostic markers in oncology have been identified over the years, including tumor grade, size, and stage. The discovery of prognostic and predictive factors is becoming increasingly relevant in medical research, especially since scientific advances have led to a greater knowledge of diseases and genetics, leading to more targeted therapy [4]. In Saudi Arabia, very few studies have been conducted to assess prognostic variables for BC patients. As a result, identifying biomarkers associated with the development of early-stage BC may aid in determining the likelihood of metastasis and guiding treatment options. An integrated bioinformatics analysis suggested that DNA replication and sister chromatid cohesion 1 (DSCC1) may act as a prognostic marker for many subtypes of BC [5]. Additionally, a previous study employing large BC cohorts and artificial neural network approaches identified *DSCC1* as one of the key genes in invasive BC samples [6].

DSCC1, also known as DCC1, is located in chromosomal region 8q24 [7]. It is a component of the selective replication factor c complex (RFC), which is involved in the S phase of the cell cycle [8]. DSCC1 and chromosome transmission-fidelity protein 18 (CTF18) combine to form a CTF18-DSCC1-CTF8 (CTF18-1-8) module. This module, along with CTF18-RFC, functions as a proliferating cell nuclear antigen loader during processes related to DNA replication [9]. Accurate chromosomal duplication and segregation are critical for genome integrity, yet stresses associated with DNA replication-related processes can promote cancer and may be key causes of genomic instability [9]. DSCC1 overexpression is reported to contribute to the growth of tumor cells in colorectal cancer, hepatocellular carcinoma, and lung cancer [7,8,10]. In addition, DSCC1 promotes tumor growth in the onset and progression of BC and may also be a potential biomarker for therapeutic intervention in BC [11]. All this evidence supports the carcinogenic activity of DSCC1 in different cancer types. However, more studies are required to comprehend the possible role of DSCC1 in various malignancies from multiple perspectives. To date, no studies have addressed the prognostic significance of DSCC1 in invasive BC and its correlation with aggressive tumor behavior. Therefore, this study aimed to explore the clinicopathological and prognostic importance of DSCC1 at the mRNA and protein levels utilizing several BC cohorts. The discovery of novel prognostic variables and therapeutic targets will help with accurate prognosis and therapeutic decisions for invasive BC.

## 2. Materials and Methods

### 2.1. Study Cohorts

The Molecular Taxonomy of Breast Cancer International Consortium (METABRIC; *n* = 1980) and The Cancer Genome Atlas (TCGA; *n* = 854) [12,13] cohorts were used to assess the correlation between *DSCC1* mRNA expression and a variety of aggressive characteristics of BC, such as molecular subtype, tumor grade, tumor size, lymphovascular invasion (LVI), and patient outcomes. To assess mRNA expression, the METABRIC cohort employed the Illumina Totalprep RNA amplification kit (Ambion, Warrington, UK) to create biotin-labeled cRNA from total RNA, which was subsequently hybridized on the Illumina Human HT-12 v3 platform. The TCGA cohort data were extracted using the Genomic Data Commons Data Portal and cBioPortal websites. The cohort was then analyzed for RNASeqV2-derived mRNA expression data [14,15].

For protein expression, 100 invasive formalin-fixed paraffin-embedded (FFPE) blocks with enough BC tumor tissue were obtained from the Histopathology Department at King Abdul Aziz Specialist Hospital (KASH). Informed consent from the respective patients was obtained. Ethical approval was obtained for using patient tissue from the Institutional Review Board at KASH (Approval Number HAP-02-T-067). Additionally, this study was conducted according to the Declaration of Helsinki. The clinicopathological profile of each patient, including histological grade, tumor size, lymph node status, LVI status, and age at diagnosis, was accessed. Estrogen receptor (ER), progesterone receptor (PR), human epidermal growth factor 2 (HER2), and Ki67 data were also available for this cohort. The ER/PR status was determined by immunohistochemistry (IHC), with tumors classified as ER+/PR+ if the staining intensity was more than 1%. Furthermore, the tumor was categorized as HER2-positive if it scored 3+ on IHC or 2+ on fluorescence in situ hybridization, indicating HER2 gene amplification [16]. Based on the St. Gallen subtypes, IHC profiles were utilized to describe the BC molecular subtypes: HER2-enriched (HER2+ independent of ER status), luminal A (ER+ and/or PR+/HER2−; Ki67 < 20%), luminal B (ER+ and/or PR+/HER2−; Ki67 ≥ 20%), and triple-negative BC (ER−, PR−, and HER2−) [16]. Outcome data were collected, including overall survival, defined as the period from diagnosis or start of treatment to death. The patients in this cohort were treated according to the National Comprehensive Cancer Network (NCCN) standards [17].

### 2.2. Immunohistochemistry for DSCC1 Protein Expression

**Figure 1:**
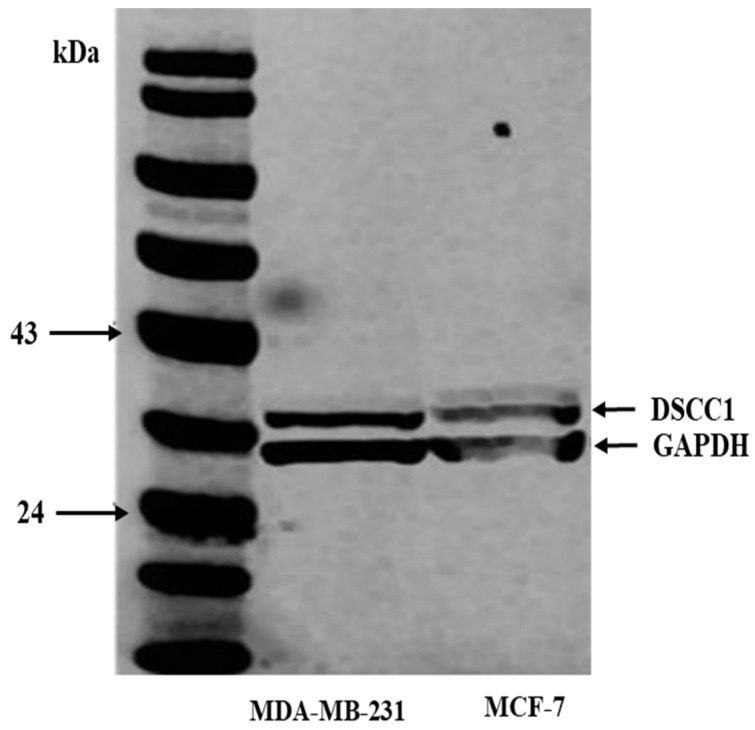
Western blot (WB) reflecting antibody specificity of DSCC1. GAPDH was used as a positive control.

The specificity of the anti-DSCC1 antibody (antibodies.com, A44495, Cambridge, UK) was confirmed by Western blotting using cell lysates of the human cell lines MDA-MB-231 and MCF-7; these were acquired from the American Type Culture Collection, Rockville, MD, USA, prior to IHC staining of the tissue sections. Fluorescent secondary antibodies at a ratio of 1:15,000 were used to detect a single band at roughly 39 kDa, following an overnight incubation at 4 °C with the rabbit anti-DSCC1 antibody (1/200; IR Dye 800CW donkey anti-rabbit and 680RD donkey anti-mouse, LI-COR Biosciences, Lincoln, UK). As a housekeeping protein, the mouse primary anti-GAPDH monoclonal antibody (ab8245, clone number 6C5, Abcam, Cambridge, UK) at 1:5000 displayed a band at around 36 kDa (Figure 1).

**Figure 2:**
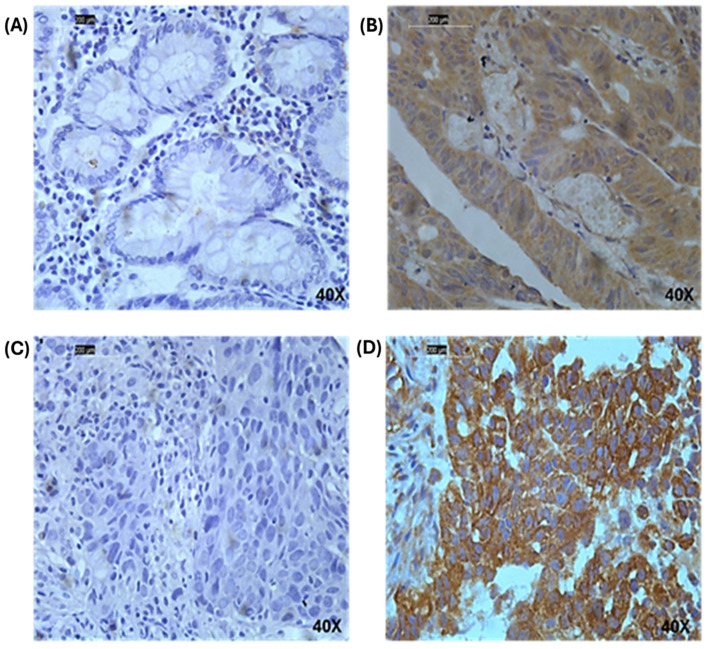
Cytoplasmic expression of DSCC1 proteins in invasive breast cancer. (**A**) Negative control of colon tissue by removing DSCC1 antibody, (**B**) positive control of colon tissue stained by DSCC1, (**C**) DSCC1-negative IHC expression, (**D**) DSCC1-positive IHC expression. Magnification, 40×; scale bars = 200 μm.

To perform IHC staining on invasive BC tissues, 4 μm tissue sections were cut with a rotary microtome (Histo-Line Laboratories, Minux^®^ S700, Sugar Land, TX, USA) and deposited on positively charged microscope slides. After being dewaxed in xylene (Fisher Scientific, X/2050, Loughborough, UK), sections were rehydrated in an alcohol series (Fisher Scientific, E/0665DF, UK) ranging from 100% to distilled water (dH_2_O). Slides were treated with 100% methanol for 15 min (Fisher Scientific, M/4056, UK) and a 0.9% hydrogen peroxide solution (H_2_O_2_, Fisher Scientific, H/1750, UK) to suppress endogenous peroxidase. Following the manufacturer’s instructions regarding antibody retrieval, microwave energy was used to retrieve the antigens (citrate buffer at a pH of 6 and at a power of 1000 W for 20 min). Sections were washed in phosphate-buffered saline (PBS) and placed in a blocking solution containing 2% (*w*/*v*) bovine serum albumin (Sigma-A4042, Haverhill, UK). A 1:10 dilution of DSCC1 primary rabbit polyclonal antibody (antibodies.com, A44495, Cambridge, UK) in the blocking buffer was added to the sections and incubated for 1 h at room temperature. Sections were then washed in PBS before being incubated in a 1:200 dilution of biotinylated anti-mouse secondary antibody in 2% BSA for about 40 min at room temperature (Vector laboratories, PK-6102, Kirtlington, UK). Before incubating the antibody with the avidin-biotin complex (ABC, Vector Labs, PK-6100, Newark, CA, USA) for 30 min at room temperature, PBS washes were performed to remove excess antibodies. Diaminobenzidine (DAB) was then added to the sections (Vector Laboratories, SK-4100, UK) after washing with PBS. The slides were washed with dH_2_O, and Mayer’s hematoxylin (Sigma, MHS16, UK) was added for counterstaining. The slides were washed with dH_2_O and immersed in several ethanol concentrations, followed by xylene. The sections were then mounted in dibutylphthalate polystyrene xylene (Sigma, 06522, UK). In IHC, negative and positive controls were included. The primary antibody was excluded from the tissue as a negative control, while a colon cancer tissue section was stained as a positive control as advised by the antibody manufacturer (Figure 2A,B).

### 2.3. DSCC1 Protein Expression Assessment

Stained sections were examined using light microscopy (Leica Microsystems, Lecia DMI 3000B, Wetzlar, Germany) at 40× magnification. Semi-quantitative assessment of DSCC1 cytoplasm expression was conducted using the modified histochemical score (H-score), for which the staining intensity was multiplied by the proportion of positive cells in each tissue. This produced a score between 0 and 300 [18]. Negative, weak, moderate, and strong scores (equivalent to a score index of 0 to 3) were used to measure intensity. The percentage of positive cells for each intensity was subjectively determined. Non-representative cores were not included in the scoring, for example, cores from invasive tumors with less than 15% core surface area or tissue that was folded during staining and processing. A professional pathologist collaborated with the lead researcher to assess IHC stained slides in a blinded and individualized way for at least 20% of the cohort under assessment. The slides were rescored in the case of a low scoring concordance, and the consultant pathologist and lead researcher reviewed the differences in the scores. Regarding DSCC1 and immunoscoring, there was a high degree of agreement between the scorers (interclass correlation coefficient [ICC] = 0.95, *p* < 0.001). Since the DSCC1 protein expression data did not follow a normal distribution, DSCC1 positivity was ascertained using the median (H-score of 130).

### 2.4. Statistical Analysis

The Statistical Package for the Social Sciences (SPSS) Version 24.0 (SPSS, Chicago, IL, USA) was used to perform statistical analysis. The concordance rate of the DSCC1 score between the two observers was ascertained using the ICC test. Data on *DSCC1* mRNA expression in the METABRIC cohort were normally distributed, and a cutoff derived from the mean was used to classify the expression levels as low or high. The TCGA cohort’s *DSCC1* mRNA expression data were right-skewed and classified using a cutoff determined by the median. The chi-square test was used to examine the association between DSCC1 and other clinicopathological features in both cohorts. Kaplan–Meier curves and the log-rank test were used in the univariate survival analysis. Multivariate analysis was conducted using the Cox regression model. All tests were deemed statistically significant if the two-tailed *p*-value was less than 0.05. The investigation was conducted in compliance with the rules provided by REMARK [19] for reporting recommendations for tumor marker prognostic studies (Supplementary Table S1).

## 3. Results

### 3.1. Transcriptomic Expression of DSCC1

A total of 839/1980 (42%) of the METABRIC BC patients and 427/854 (50%) of the TCGA cohort cases had high *DSCC1* mRNA expression. In both cohorts, a significant correlation was observed between high *DSCC1* mRNA expression and large tumor size, high tumor grade, LVI positivity, hormone receptor negativity (ER−/PR−; all *p* < 0.001), and HER2 positivity (*p* < 0.001 in METABRIC and *p* = 0.027 in TCGA). In the METABRIC cohort, a high *DSCC1* mRNA level was significantly associated with young age (*p* = 0.013), premenopause (*p* = 0.004), a poor Nottingham prognostic index (*p* < 0.001), and lymph node stage (*p* = 0.002).

Regarding the intrinsic (PAM50) subtypes, high expression of *DSCC1* mRNA was found in cases with basal-like, HER2-enriched luminal B, luminal A, and normal-like subtypes in descending order (*p* < 0.001; Table 1).

### 3.2. Proteomic Expression of DSCC1

The DSCC1 protein was expressed in the cytoplasm of invasive breast tumor tissues, with no discernible membranous or nuclear staining and with varying degrees of intensity, from absent to high (Figure 2C,D). High DSCC1 protein expression (H-score >130) was observed in 48/100 (48%) of invasive BC cases. The correlation between DSCC1 protein expression and clinicopathological parameters in the KASH cohort was investigated. High DSCC1 protein expression was significantly associated with high tumor grade (*p* < 0.001), negative hormone receptor (ER−: *p* = 0.007, PR−: *p* = 0.005), and high Ki67 (*p* = 0.036). In the IHC molecular subtypes, high expression of DSCC1 was associated with the triple-negative, HER2+, and ER+/HER2− high-proliferation subtypes, followed by the ER+/HER2− low-proliferation subtype (*p* = 0.005; Table 2).

### 3.3. DSCC1 Expression and Patient Outcomes

**Figure 3:**
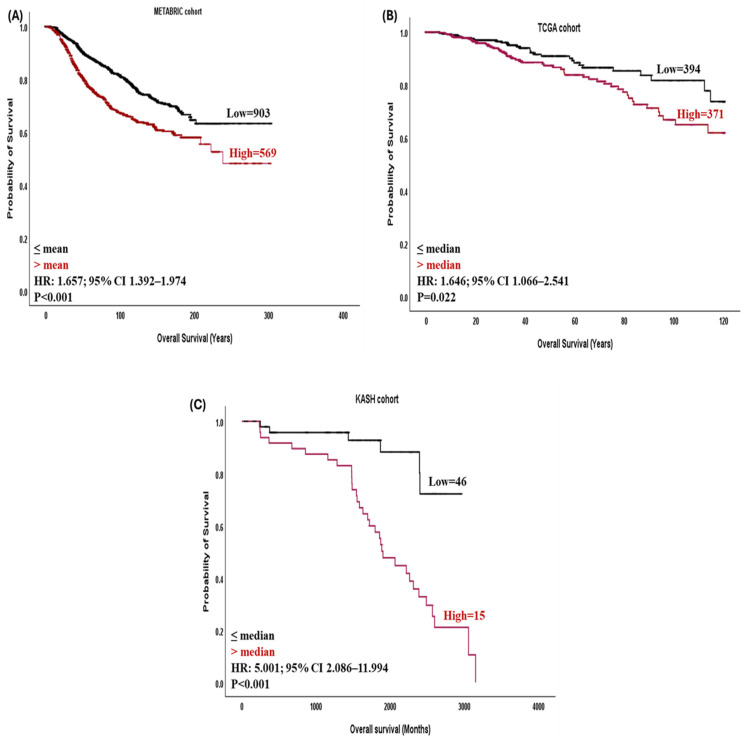
Kaplan–Meier survival plots showing the association between DSCC1 mRNA expression and overall survival in (**A**) the whole METABRIC cohort, (**B**) the whole TCGA cohort, and (**C**) the whole KASH cohort.

In both the METABRIC and TCGA cohorts, high expression of *DSCC1* mRNA was significantly associated with poor outcomes (*p* < 0.001 in METABRIC and *p* = 0.022 in TCGA; Figure 3A,B). In the univariate analysis at the protein level, high DSCC1 expression was correlated significantly with poor patient outcomes (*p* < 0.001; Figure 3C). Cox regression analysis of the METABRIC and KASH cohorts showed that high DSCC1 expression was a significant predictor of shorter survival regardless of tumor size, lymph node status, and tumor grade (hazard ratio (HR) = 1.325; 95% confidence interval (CI) 1.098–1.599; *p* = 0.003 in METABRIC and HR = 6.381; 95% CI 1.489–27.339; *p* = 0.013 in KASH; Table 3).

## 4. Discussion

BC is well recognized as a heterogeneous and complex condition, with various subgroups having distinct prognoses [20]. This complexity presents difficulty in precisely understanding the biology of BC and, hence, defining the therapeutic approach to the disease [2]. Identifying biomarkers linked to the onset of early-stage BC could aid in determining the likelihood of metastasis and guiding treatment options. DCSS1 was previously found to be a principal gene in invasive BC [6]. Several preclinical studies have demonstrated the potential role of DSCC1 in cancer progression in many cancers, including gastric cancer [21], lung adenocarcinoma [8], and BC [11]; however, this is the first study to conduct a comprehensive analysis of the correlation between DSCC1 and clinicopathological parameters and patient outcomes at the transcriptomic and protein levels. The clinicopathological and prognostic importance of DSCC1 in BC remains unknown, and this study aimed to address this gap.

The significance of DCCS1 in carcinogenesis has received more attention in recent years. Previous investigations have shown that colorectal cancer, lung adenocarcinoma, and hepatocellular carcinoma cells display higher levels of DSCC1 [7,22]. DSCC1 may contribute to oncogenesis by influencing DNA repair, apoptosis, and the proliferation of multiple cells. Human cancer is characterized by aberrant cell cycle control and abnormalities in DNA replication [23]. DSCC1 was shown to increase the metastasis of lung adenocarcinoma cells by increasing their capacity for cell division, stemness, and epithelial–mesenchymal transition (EMT). EMT is a critical phase in the early stages of cancer metastasis and is controlled by signaling pathways such as STAT3 and Smad2/3. DSCC1 siRNA inhibited the progression of EMT in lung adenocarcinoma cells by increasing E-cadherin and decreasing N-cadherin [24]. DSCC1 expression also has a substantial positive association with the presence of immune cell types (CD8+ T cells, CD4+ T cells, and B cells), possibly altering the tumor microenvironment and leading to cancer development [25]. All these previous findings demonstrate the potential role of DSCC1 in cancer biology.

Reduced DNA methylation can cause increased RNA transcription. Using the OncoDB database, a previous study indicated that DSCC1 promoter methylation in certain cancers, such as kidney renal papillary cell carcinoma and hepatocellular carcinoma, was considerably reduced compared to that in equivalent normal tissues, indicating a mechanism for promoting RNA transcription [25]. Additionally, cancer incidence and progression are closely connected to genetic alterations. To determine the mutational features of DSCC1, cBioPortal was used in a previous study for comparative analysis. The results showed that DSCC1 was altered in 17% of liver hepatocellular carcinoma samples and 5% of lung adenocarcinoma samples, indicating the role of genetic alterations in the dysregulation of DSCC1 [8,25]. In vivo investigations have shown that E2F4 promotes tumor development and metastasis in nude mice. Notably, E2F4 is a critical transcriptional regulator of DSCC1, activating it by binding to particular locations in the DSCC1 promoter region. DSCC1 was also discovered to enhance malignancy in gastric cancer, namely cell proliferation, both in vitro and in vivo [21].

The results of *DSCC1* evaluation in this study revealed that DSCC1 is highly expressed in invasive BC and correlated with aggressive features of the tumor at the mRNA and protein levels. High expression of DSCC1 was significantly associated with large tumor size, high tumor grade, ER negativity, PR negativity, and high levels of Ki67. This supports the results of a previous pan-cancer study showing that DSCCI is overexpressed in tumor tissues compared to non-tumor tissues, e.g., in colorectal cancer and hepatocellular carcinoma [22]. These findings add to the evidence supporting the potential role of DSCC1 in tumor growth. Furthermore, DSCC1 mRNA and protein were shown to be substantially elevated in triple-negative and HER2-positive BC. Triple-negative BC is highly malignant and prone to metastasis and recurrence and is linked with limited treatment options and a poor prognosis compared to other BC subtypes [26]. High DSCC1 expression may promote the activity of the HER2 signaling pathway, which can trigger the tumor microenvironment to promote tumor cell proliferation, invasion, and metastasis [27,28]. These results imply that DSCC1 may be a crucial gene, has an oncogenic impact, and contributes to carcinogenesis in BC.

This study highlighted that high expression of DSCC1 was associated with young age and the lymph nodal stage, which may influence BC prognosis. This is supported by previous findings indicating that genetic factors may affect BC prognosis. BC is more likely to manifest at a younger age (≤35 years) and have more aggressive traits, including being grade 3, triple-negative, and Ki67 ≥ 25%, in young women with BRCA mutations than those without mutations. A previous study demonstrated that young BRCA-mutation carriers exhibited a poorer prognosis for recurrence and survival than non-carriers [29]. In addition, the lymph nodal stage is a critical predictor of the prognosis of BC patients. Sentinel lymph node biopsy is linked to better long-term results, including recurrence-free survival, distant disease-free survival, overall survival, and breast cancer-specific survival, than axillary lymph node dissection [30].

The association between Ki67 and the high level of DSCC1 found in this study demonstrates DSCC1’s prognostic significance. Ki67 expression is established as a marker of oncogenesis that is closely associated with aggressive tumor characteristics, proliferation, and reduced survival rates [31]. Furthermore, DSCC1 expression may be elevated in specific tumor types. This may pertain to tumor cells’ increased need for DNA replication and cellular proliferation. Research indicates that DSCC1 is significantly expressed in lung adenocarcinoma and associated with an unfavorable prognosis. DSCC1 is also significantly upregulated in hepatocellular carcinoma, where it facilitates tumor cell proliferation [23]. A study also demonstrated that when cytoplasmic DSCC1 expression is elevated in the area of a tumor, colon cancer patients’ probability of survival decreases. DSCC1 has been investigated as a potential tumor biomarker for early diagnosis and prognosis in a few studies. By disrupting DSCC1’s functions, tumor cells’ proliferation and survival may be influenced [7,23]. Therefore, DSCC1 is a significant prognostic biomarker in BC

This study also found that DSCC1 was correlated with poor prognostic features and short-term survival outcomes in BC. High DSCC1 expression was found to be an independent predictor of shorter survival in both proteomic and transcriptome datasets. This finding was consistent with a previous study showing a correlation between the high expression of DSCC1 and poor outcomes in hepatocellular carcinoma at the mRNA and protein levels [22]. Our results also support a previous in vitro functional investigation of DSCC1, showing that DSCC1 promotes the proliferation, invasion, and migration of BC cells, possibly by activating the Wnt/β-catenin pathway and modulating p53 levels [11]. GSEA analysis of the public TCGA dataset of BC samples revealed a significant correlation between elevated DSCC1 expression, the p53 and Wnt pathways, and the cell cycle. In malignancies, P53 is a recognized inhibitor that modulates the cell cycle and mitigates proliferation in BC. This suggests that the expression of DSCC1 and p53 should be inversely correlated in BC, which has been confirmed empirically [11,32]. The Wnt/β-catenin pathway also plays a significant role in BC. It comprises multiple essential components, including GSK-3b and β-catenin. β-catenin is a crucial downstream molecule of the Wnt pathway and a significant protein in BC recurrence and metastasis. GSK-3b is a vital element of the β-catenin degradation complex, and its phosphorylation inactivates this complex in tumor cells [33]. Cyclin D1 is a significant downstream target of the Wnt/β-catenin pathway that is closely associated with β-catenin. A study showed that DSCC1 was positively associated with p-GSK-3b, β-catenin, and cyclin D1 but negatively correlated with p-β-catenin [34]. DSCC1 reportedly stimulates the Wnt/β-catenin pathway and suppresses p53 levels, promoting BC development [11]. However, as the Wnt/β-catenin pathway involves several molecules, the precise mechanism by which DSCC1 promotes BC development merits further investigation. The association between the high expression of DSCC1in this study and poor prognosis factors emphasizes its correlation with cancer onset and metastasis.

All these findings suggest that *DSCC1* is a prognostic and therapeutic target and may play a critical role in the metastatic process. The findings, which support previous research, show that DSCC1 correlates with aggressive clinicopathological characteristics in invasive BC, which may lead to metastatic processes such as invasion and migration. More studies are needed to determine its potential as a therapeutic target and investigate how *DSCC1* regulates critical downstream biological processes and signaling to govern tumor initiation and development. Despite the remarkable findings of this study, one limitation is the number of samples used in the proteomic analysis. Although the number is acceptable, the sample offers suitable statistical power. Further study with a larger sample size will affirm the significance of these findings. More research is needed to determine its potential as a therapeutic target and investigate how *DSCC1* regulates critical downstream biological processes and signaling to govern tumor initiation and development

## 5. Conclusions

The predictive importance of DSCC1 in invasive BC patients was validated by this investigation. In BC, DSCC1 may be a therapeutic target for cases with unfavorable outcomes.

## Figures and Tables

**Table 1:** Statistical associations between *DSCC1* mRNA expression and clinicopathological parameters in the METABRIC (*n* = 1980) and TCGA (*n* = 854) breast carcinoma datasets.

Parameters	*DSCC1* mRNA (METABRIC)			*DSCC1* mRNA (TCGA)		
	Low (N = 1141) N. (%)	High (N = 839) N. (%)	χ^2^ (*p*-Value)	Low (N = 427) N. (%)	High (N = 427) N. (%)	χ^2^ (*p*-Value)
**Patient age (years)**						
≤50	222 (42)	202(48)	6.131	108 (47)	123 (53)	1.335
>50	919 (59)	637 (41)	**(0.013)**	319 (51)	304 (49)	(0.248)
**Menopausal status**						
Premenopausal	225 (52)	221 (48)	8.078	Not available		
Postmenopausal	908 (59)	625 (41)	**(0.004)**			
**Tumor size (cm)**						
≤2 cm	393 (63)	229 (37)	11.414	147 (62)	92 (38)	17.576
>2 cm	737 (55)	601 (45)	**(<0.001)**	280 (46)	335 (54)	**(<0.001)**
**Tumor grade**						
I	135 (79)	35 (21)		77 (87)	12 (13)	
II	563 (73)	207 (27)	221.909	261 (70)	114 (30)	240.076
III	386 (40)	566 (60)	**(<0.001)**	67 (19)	285 (81)	**(<0.001)**
**Nottingham prognostic index**						
Good	506 (74)	174 (26)				
Moderate	543 (49)	558 (51)	120.162	Not available		
Poor	92 (46)	107 (54)	**(<0.001)**			
**Lymph node stage**						
I	637 (62)	398 (38)				
II	333 (54)	289 (46)	12.995	Not available		
III	169 (54)	147 (46)	**(0.002)**			
**Lymphovascular invasion**						
Negative	585 (63)	345 (37)	27.303	320 (57)	239 (43)	33.978
Positive	315 (50)	320 (50)	**(<0.001)**	107 (36)	188 (64)	**(<0.001)**
**Estrogen receptor (ER)**						
Negative	173 (37)	301 (63)	113.929	29 (16)	156 (84)	117.270
Positive	968 (64)	538 (36)	**(<0.001)**	389 (61)	250 (39)	**(<0.001)**
**Progesterone receptor (PR)**						
Negative	448 (48)	492 (52)	72.802	71 (26)	201 (74)	98.908
Positive	693 (67)	347 (33)	**(<0.001)**	344 (63)	202 (37)	**(<0.001)**
**Human epidermal growth factor receptor 2 (HER2)**						
Negative	1040 (60)	693 (40)	32.369	295 (52)	272 (48)	4.910
Positive	101 (41)	146 (59)	**(<0.001)**	55 (41)	78 (59)	**(0.027)**
**PAM50 subtype**						
Luminal A	574 (80)	144 (20)				
Luminal B	206 (42)	282 (58)				
HER2+-enriched	94 (39)	146 (61)	423.455	Not available		
Basal-like	90 (27)	239 (73)	**(<0.001)**			
Normal-like	174 (87)	25 (13)				

*p*-values in bold are statistically significant.

**Table 2:** Statistical associations between DSCC1 protein expression and clinicopathological factors in the KASH breast cancer cohort (n = 100).

Clinicopathological Parameters	DSCC1 Protein Expression		χ^2^ (*p*-Value)
	Low (N = 50) N. (%)	High (N = 50) N. (%)	
**Patient age (years)**			
≤50	23 (49)	24 (51)	0.334
>50	29 (55)	24 (45)	(0.564)
**Menopausal status**			
Pre-menopausal	23 (49)	24 (51)	0.334
Post-menopausal	29 (55)	24 (45)	(0.564)
**Tumor size (mm)**			
≤10 mm	17 (55)	14 (45)	0.010
>10 mm	15 (54)	13 (46)	(0.922)
**Tumor grade**			
I	8 (100)	0 (0)	
II	38 (70)	18 (32)	42.270
III	1 (3)	29 (97)	**(<0.001)**
**Lymph-vascular invasion**			
Negative	8 (36)	14 (64)	2.277
Positive	13 (59)	9 (41)	(0.131)
**Oestrogen receptor (ER)**			
Negative	6 (27)	16 (73)	7.233
Positive	46 (60)	31 (40)	**(0.007)**
**Progesterone receptor (PR)**			
Negative	7 (28)	18 (72)	8.068
Positive	45 (61)	29 (39)	**(0.005)**
**Human epidermal growth factor receptor 2 (HER2)**			
Negative	37 (51)	36 (50)	0.211
Positive	14 (56)	11 (44)	(0.646)
**Ki67**			
Low	25 (66)	13 (34)	4.398
High	25 (44)	32 (56)	**(0.036)**
**Immunohistochemistry subtype**			
ER+/HER2- low proliferation	27 (69)	12 (31)	
ER+/HER2- high proliferation	18 (50)	18 (50)	
Triple-negative	2 (14)	12 (86)	12.688
HER2+	4 (50)	4 (50)	**(0.005)**

*p*-values in bold are statistically significant.

**Table 3:** Multivariate Cox regression analysis for predictors of overall survival and DSCC1 mRNA expression in the METABRIC and TCGA cohorts and protein expression in the KASH cohort.

Parameters	Hazard Ratio (HR)	95% Confidence Interval (CI)		*p*-Value
		Lower	Upper	
METABRIC cohort (mRNA)				
*DSCC1* mRNA expression	1.325	1.098	1.599	**0.003**
Tumor size	1.596	1.278	1.993	**<0.001**
Lymph node	2.231	1.842	2.702	**<0.001**
Tumor grade	1.403	1.192	1.652	**<0.001**
TCGA cohort (mRNA)				
*DSCC1* mRNA expression	1.580	0.920	2.712	0.097
Tumor size	1.419	0.814	2.473	0.217
Lymph node	1.656	1.042	2.632	**0.033**
Tumor grade	1.114	0.755	1.644	0.586
KASH cohort (protein)				
DSCC1 protein expression	6.381	1.489	27.339	**0.013**
Tumor size	0.686	0.267	1.763	0.434
Lymph node	1.059	0.395	2.840	0.910
Tumor grade	0.577	0.229	1.456	0.244

*p*-values in bold are statistically significant.

## Data Availability

Data are unavailable due to privacy or ethical restrictions. Data cannot be provided without the permission of the Directorate of Health Affairs.

## Supplementary Materials

The following supporting information can be downloaded at: https://www.mdpi.com/article/10.3390/medicina60121929/s1, Table S1: PREMARK criteria for the study.
